# A2 β-casein infant formula with high sn-2 palmitate and casein phosphopeptides (CPP) supports energy and mineral absorption in Chinese infants

**DOI:** 10.3389/fnut.2026.1771135

**Published:** 2026-03-10

**Authors:** Li Wang, Wiola Mi, Hai Rong Liu, Nan Sheng, Hui Dong Huang, Xiao Jiang Jia, Xiao Yang Sheng

**Affiliations:** 1 Bunge Nutrition, Shanghai, China; 2 Junlebao Nutrition Research Institute, Shijiazhuang, China; 3 Department of Developmental Behavioral Pediatric and Children Healthcare, Xinhua Hospital, School of Medicine, Shanghai Jiao Tong University, Shanghai, China

**Keywords:** A2 β-casein, calcium absorption, casein phosphopeptides (CPP), fecal fatty acid soaps, infant nutrition, mineral absorption, sn-2 palmitate

## Abstract

Infant formula (IF) is essential when breastfeeding is not possible or sufficient. We previously reported that, in a randomized, double-blind clinical trial with 180 healthy infants, an A2 β-casein IF containing high sn-2 palmitate levels, and casein phosphopeptides (CPP) improved stool consistency, gastrointestinal comfort, crying time, and bone mineral density. Here, we present complementary, pre-specified, biochemical analyses from the same cohort. Relative to standard cow’s milk IF, the A2 β-casein, high sn-2 palmitate and CPP IF reduced fatty acid and calcium excretion, reduced calcium fatty acid soap formation, increased hair iron and zinc content, and was associated with lower cortisol levels. These parameters were not different from the breastfed group in the study. Together, the previously published clinical outcomes and the biochemical findings presented here suggest that an IF designed to narrow the compositional gap with human milk may deliver some of the advantages of breastfeeding, providing a path for improving IF compositions.

## 1 Introduction

Human milk (HM) delivers the ideal blend of nutrients and bioactive components tailored to meet infant needs. Nonetheless, there are scenarios when breastfeeding may not be possible, leading to the adoption of IFs (1). Fat, the second most abundant macronutrient in both HM and IF, provides nearly 50% of an infant’s total energy needs and is essential for nervous system development (2). In HM, up to 98% of the lipids are triglycerides (3) containing both saturated and unsaturated fatty acids. Palmitic acid, the primary saturated fatty acid in HM, accounts for about 20%–25% of total fatty acids and 60%–70% of it is esterified at the triglyceride’s sn-2 position (4). However, most IFs rely on vegetable oils as their fat source, where palmitic acid is predominantly in sn-1 or sn-3 positions, and less than 20% is present at sn-2 (5). Studies in both pre-term and term infants have investigated the effects of palmitic acid positioning, showing that sn-2 palmitate enhances fatty acids and calcium absorption (6–9) and reduces the formation of fatty acid calcium soaps in the gut, resulting in softer stools which, in turn, lead to increased gut comfort, less crying, and better sleep (10–15).

Milk proteins are another critical component of HM and IF. In cow’s milk, protein is comprised of whey and casein fractions. β-casein, making up to 45% of total caseins, is a phosphorylated protein that can be broken down into bioactive peptides, influencing digestive, metabolic, hormonal, immune, and neural health (16). Cow’s milk typically includes two variants of β-casein, A1 and A2, while HM exclusively contains A2 β-casein (17). A1 β-casein undergoes hydrolysis to form β-casomorphin-7 (BCM-7), which activates gastrointestinal (GI) opioid receptors, impacting the endocrine and immune systems and leading to decreased GI motility and prolonged GI transit time (16). Consequently, A1 β-casein has been linked to GI intolerance. In contrast, A2 β-casein does not lead to BCM-7 production, suggesting that milk containing only A2 β-casein may result in improved GI motility and fewer GI symptoms which result in reduced stress and inflammatory markers (18, 19). This distinction has been supported by animal studies (18), and human trials involving toddlers (20), preschoolers (21), and infants (15).

Calcium (Ca), iron (Fe) and zinc (Zn) are micronutrients crucial for optimal infant growth, development, and health (22–24); they play pivotal roles in numerous enzyme systems (25). Infants undergoing critical growth phases are particularly vulnerable to micronutrient deficiencies (26, 27). Hence, ensuring adequate intake, absorption, and bioavailability of calcium, iron, and zinc is paramount in IF development. Studies have demonstrated that IFs enriched in sn-2 palmitate enhance calcium absorption, increase bone mineralization, and reduce the formation of calcium soaps in stools (15, 28). In breast-fed infants, casein phosphopeptides are naturally generated in the infant gut through the digestion of β-casein present in HM. To mimic the effect of HM-derived phosphopeptides, CPP derived from bovine milk protein digestion is added to IF to acts as mineral carrier, enhancing bioavailability (29). CPP possesses highly polar, acidic sequences that attach to minerals preventing precipitation in the intestine and aiding in their transport. While CPP have shown positive effects on calcium, iron, and zinc uptake in studies involving Caco2 cells and rats (30, 31), to our knowledge, our recent study is the only one that investigated an IF supplemented with both high sn-2 palmitate and CPP. In that study, infants fed the investigational formula showed improved bone structure with adequate growth and development when compared to conventional IF, results consistent with enhanced mineral uptake (15).

Considering the beneficial effects on digestive health and bone structure shown in our randomized, double-blind, controlled clinical trial investigating an A2 β-casein IF with high sn-2 palmitate and CPP (15), this work aims at providing biochemical support to those results. Biological samples collected from the subjects in that study were subject to pre-specified biochemical analyses measuring fecal fatty acids, calcium excretion and fecal calcium soaps, hair iron and zinc levels, and stress and immune markers.

## 2 Materials and methods

### 2.1 Study participants and study design

Double-blind, randomized, controlled trial (Clinicaltrials.gov: NCT04749290) with 180 infants at a clinical site in Jin Hua, Zhejiang Province, China (15). Infants were assigned to three groups: (1) investigational (A2 β-casein cow’s milk IF fortified with high sn-2 palmitate and CPP; *n* = 60), (2) control (standard cow’s milk IF with low sn-2 palmitate and no CPP; *n* = 60), or (3) HM (exclusively breastfed) (*n* = 60). See (15) for details.

### 2.2 Sample collection

Sample analyses were pre-specified in the clinical study protocol. However, as the clinical study started in March 2020 and the biochemical analyses presented in this work were performed after study completion, only samples that have been stored for less than 2 years were analyzed. To ensure results’ integrity, a strict blinding protocol was maintained. Biological samples were de-identified and labeled solely with unique subject codes. Laboratory analysts and technicians were blinded to interventions and subject identities.

Stool: Samples were collected at home by parents or caregivers using anal swabs and immediately stored at −20°C. Stool samples for palmitic acid, stearic acid, and calcium excretion were collected at 30, 60, 90, 120, and 150 days of age. Stool samples for fatty acid soaps were collected at 30 and 150 days.

Hair: Samples were cut ∼1 cm from the scalp using stainless steel scissors, sealed in labeled polyethylene bags, and stored at room temperature. Hair samples were collected at 30, 90, and 150 days.

Saliva: Samples were collected using the SalivaBio Infant’s Swab (SIS), held securely on the tongue for 60–90 s or until saturation. If needed, swabs were reintroduced until adequate volume was obtained. Samples were centrifuged, transferred to storage tubes, and frozen at −20 °C. Saliva samples were collected at 30 and 150 days.

### 2.3 Palmitic and stearic acids analysis

Fecal palmitic and stearic acids were determined by GC-MS following Ge et al. (32) with modifications. Briefly, fecal samples were homogenized in dichloromethane-methanol (2:1 v/v) and washed with Milli-Q water (Optima™ LC/MS Grade, Fisher Chemical™). The organic phase was collected, dried under nitrogen, and reconstituted in 2 ml of n-hexane. After addition of an internal standard, samples were methylated, phase-separated, and the hexane layer evaporated and redissolved for GC-MS analysis.

Analyses were performed on an Agilent 5977B MSD coupled to a GC with HP-5ms column (30 m × 250 μm × 0.25 μm). Temperature program: initial temperature of 80 °C, ramped to 180 °C at 20 °C/min (8 min hold), then to 280 °C at 5 °C/min (3 min hold). Helium served as the carrier gas (1.0 ml/min). System stability and repeatability were monitored with quality control samples. MS settings: inlet at 280 °C, ion source at 230 °C, transfer line at 250 °C, electron impact ionization at 70 eV, operated in combined SCAN/SIM mode.

### 2.4 Fecal calcium and calcium soaps analysis

Fecal soap fatty acids were analyzed following a method adapted from Quinlan et al. (33). Briefly, neutral lipids from freeze-dried and powdered feces (500 mg) were extracted by refluxing with petroleum ether (boiling point, 30 °C–60 °C). The residue was acidified with acetic acid to release soap fatty acids, which were re-extracted with petroleum ether. C17:0 (Sigma-Aldrich Co., Ltd., Shanghai, China) was used as an internal standard. Non-soap fatty acids were isolated by preparative TLC (n-hexane/diethyl ether/formic acid, 70:30:1, v/v/v), converted to methyl esters using boron trifluoride/methanol, and quantified by GC (34). Total fecal fatty acids were the sum of soap and non-soap fatty acids.

Fecal calcium concentration was determined using a colorimetric assay kit (Elabscience Biotechnology Co., Ltd., Wuhan, China) (34). Samples were homogenized in PBS (0.01 M, pH = 7.4) at a ratio of 1:9 w/v, centrifuged (5000 rpm for 10 min at 4 °C) and 10 μl of the supernatant was mixed with the MTB, alkali, and clarifying reagents (10:20:1 v/v/v), mixed (vortex for 30 s), and incubated at 37 °C for 5 min. Absorbance was measured at 610 nm.

### 2.5 Measurement of hair trace elements

Prior to analysis, hair samples were washed with acetone and deionized water to remove external contaminants, ensuring that the measured values reflect endogenous trace element levels. 10 mg of hair was ground with six 3-mm steel balls for 360 s at 60 Hz at room temperature. The resulting powder was homogenized in 100 μl of HAc-NaAc on ice and centrifuged at 12,000 rpm for 5 min at 4 °C. The supernatant was collected and kept on ice until analysis. Total iron concentration was determined using a Ferrozine colorimetric assay (RXFG0460-48, Ruixing Biotech, China) following acidolysis. Zinc levels were measured using a direct colorimetric assay, following the Di-Br-PAESA method (RXDC7034, Ruixing Biotech, China).

### 2.6 Stress and immunity markers analysis

Salivary cortisol and TNF-α were quantified by ELISA (MM-0974H1 and MM-0122H1, Jiangsu Enzyme Immunoassay Industry Co., Ltd., China).

### 2.7 Statistical analyses

Statistical analyses were conducted with SAS v9.4 (SAS Institute, Cary, NC, USA), using two-tailed Kruskal-Wallis (fecal palmitic and stearic acids, and fecal fatty acid calcium soaps) or two-tailed ANOVA (Ca excretion, hair trace elements, stress, and immune markers) with a significance threshold of 0.05. Pairwise comparisons among Investigational, Control, and HM groups were adjusted for multiple comparisons using the Bonferroni correction.

## 3 Results

### 3.1 Subject characteristics

A total of 180 participants were enrolled and evenly distributed across the three study groups (*n* = 60 each). The groups showed no significant differences in age, sex, birth history, or primary caregiver. Feeding habits prior to enrollment were similarly balanced between the two formula groups.

### 3.2 Palmitic and stearic acid excretion

**FIGURE 1:**
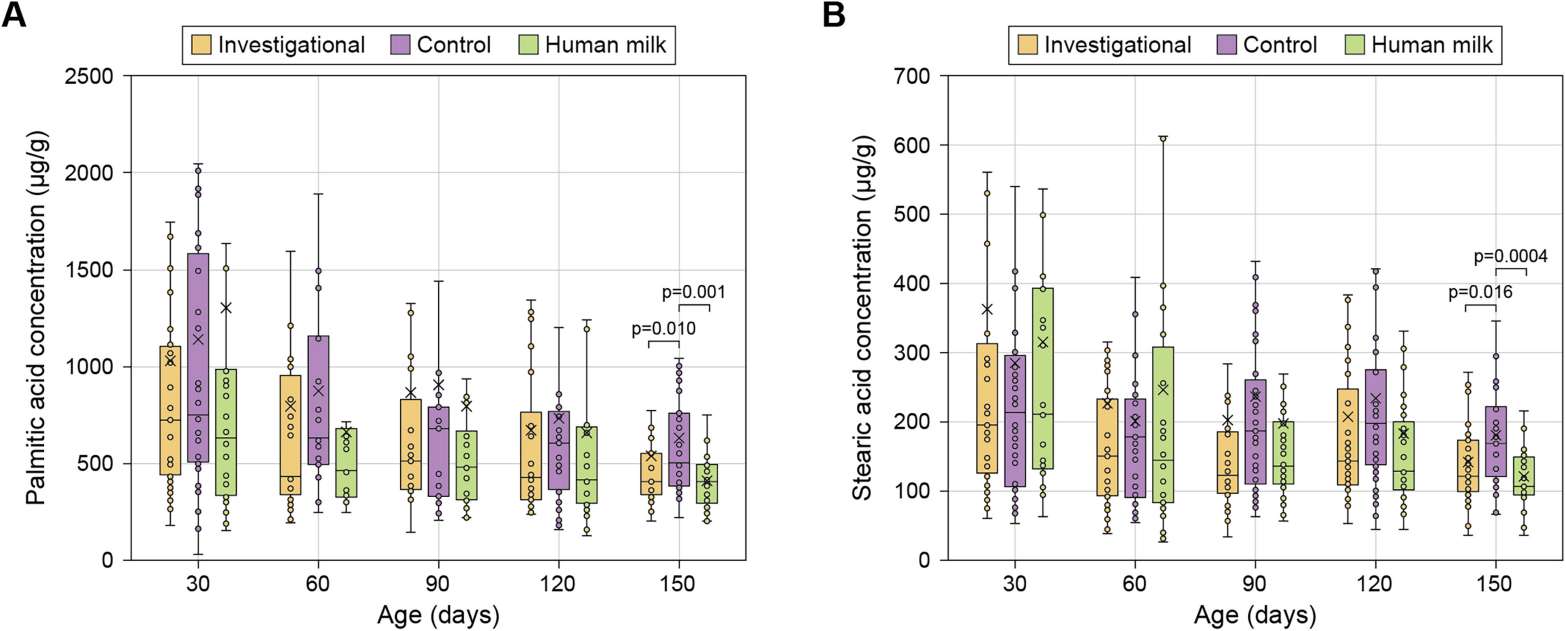
Fecal palmitic acid **(A)** and stearic acid **(B)**. Data are shown as medians (horizontal bar), IQRs (box), ranges (whiskers); arithmetic means are also indicated (cross). See Table 1 for sample sizes.

Fecal palmitic and stearic acids levels, indicators of fatty acid and energy absorption, were measured between 30 and 150 days in the three groups. By 150 days, the Investigational and HM groups showed similar levels of fecal palmitic and stearic acids, but those levels were significantly lower than the Control group (19% and 20% lower palmitic acid, and 28% and 37% lower stearic acid for Investigational and HM groups, respectively). No differences were observed at earlier time points (30–120 days) (Figure 1 and Table 1).

**TABLE 1:** Data and statistical analyses.

Continuous variables	Age	Investigational		Control		Human milk		*P*-value		
	(days)	Value	*n*	Value	*n*	Value	*n*	I vs. C	I vs. HM	C vs. HM
**Fecal fatty acids**										
Palmitic acid^1^	30	724.31 (433.22, 1114.85)	35	750.31 (500.06, 1612.01)	42	631.84 (334.19, 991.20)	28	0.709	0.871	0.604
	60	622.98 (332.70, 998.02)	39	725.53 (453.19, 1169.59)	35	584.32 (349.74, 819.95)	30	0.156	0.857	0.248
	90	503.29 (362.09, 711.77)	40	680.64 (333.58, 853.68)	35	483.61 (313.96, 667.98)	30	0.764	0.660	0.481
	120	441.39 (312.86, 1104.60)	39	604.91 (356.00, 791.89)	35	414.96 (289.80, 698.78)	31	0.632	0.618	0.349
	150	411.01 (335.02, 551.40)	39	506.56 (383.70, 762.52)	35	406.87 (286.42, 498.57)	31	*0.010*	0.288	*0.001*
Stearic acid^1^	30	196.34 (124.94, 328.59)	35	213.62 (105.38, 301.36)	42	212.12 (132.80, 393.62)	28	0.647	0.949	0.620
	60	150.58 (92.21, 234.03)	39	179.23 (86.95, 239.94)	35	145.16 (83.58, 326.75)	30	0.834	0.965	0.878
	90	123.16 (96.40, 188.22)	40	187.12 (105.49, 269.92)	35	136.90 (110.13, 201.75)	30	0.101	0.759	0.217
	120	143.85 (108.31, 270.18)	39	198.02 (136.33, 279.88)	35	128.62 (96.80, 209.95)	31	0.227	0.449	0.062
	150	121.23 (100.18, 174.38)	39	169.49 (122.01, 222.22)	35	107.51 (95.35, 149.14)	31	*0.016*	0.162	*0.0004*
**Calcium excretion and fecal calcium soaps**										
Ca (μg/g)^2^	30	18.76 ± 4.69	37	19.54 ± 4.20	42	18.95 ± 3.45	28	0.412	0.862	0.562
	60	18.99 ± 4.28	39	19.29 ± 3.87	35	18.34 ± 3.32	31	0.741	0.484	0.321
	90	18.22 ± 4.31	39	19.26 ± 4.02	35	17.98 ± 3.77	31	0.275	0.808	0.205
	120	18.18 ± 3.38	39	19.22 ± 3.62	35	17.37 ± 3.46	31	0.205	0.373	0.042
	150	16.81 ± 3.63	39	19.17 ± 4.37	35	16.71 ± 3.43	31	*0.010*	0.915	*0.011*
Total fatty acid Ca soaps (μg/g)^1^	30	6873.53 (4089.26, 11659.69)	35	6713.65 (5282.77, 9819.85)	42	6318.23 (4866.62, 9805.63)	28	0.821	0.780	0.615
	150	4523.56 (2911.4, 7658.61)	39	5702.07 (4388.11, 9447.9)	35	3917.7 (3247.34, 6514.99)	31	*0.013*	0.883	*0.012*
**Hair trace elements**										
Fe (μg/g)^2^	30	8.13 ± 2.08	16	8.24 ± 2.92	18	8.21 ± 2.61	16	0.903	0.929	0.976
	90	10.40 ± 3.03	14	10.11 ± 2.09	16	10.48 ± 2.98	15	0.770	0.938	0.705
	150	16.75 ± 2.96	14	13.84 ± 2.83	17	16.96 ± 3.84	15	*0.016*	0.861	*0.010*
Zn (μmol/g)^2^	30	48.50 ± 15.18	16	54.16 ± 17.47	18	50.98 ± 18.76	16	0.344	0.686	0.593
	90	72.33 ± 14.07	14	66.84 ± 16.70	16	75.16 ± 16.88	15	0.354	0.637	0.155
	150	94.74 ± 15.52	14	80.18 ± 12.69	17	97.47 ± 16.50	15	*0.01*	0.624	*0.002*
**Stress and immunity markers**										
Cortisol (ng/L)^2^	30	220.77 ± 19.6	32	228.13 ± 21.71	39	221.28 ± 24.58	28	0.162	0.929	0.210
	150	201.90 ± 18.11	49	212.44 ± 20.52	50	200.84 ± 18.31	50	*0.007*	0.782	*0.003*
TNF-α (μg/L)^2^	30	462.40 ± 38.15	32	459.59 ± 48.84	39	446.48 ± 52.89	28	0.784	0.285	0.393
	150	417.55 ± 41.91	49	428.50 ± 37.61	50	412.86 ± 39.32	50	0.171	0.557	0.050

^1^Data are summarized as median and IQR (1st quartile, 3rd quartile). Group differences were evaluated using Kruskal-Wallis’s test. Significance level for multiple comparisons was 0.05/3 = 0.017 with Bonferroni correction.

^2^Data are summarized as mean ± standard deviation. Group differences were evaluated using analysis of variance (ANOVA). Significance level for multiple comparisons was 0.05/3 = 0.017 with Bonferroni correction. Significant comparisons italicized.

### 3.3 Calcium excretion and fecal fatty acid calcium soaps

**FIGURE 2:**
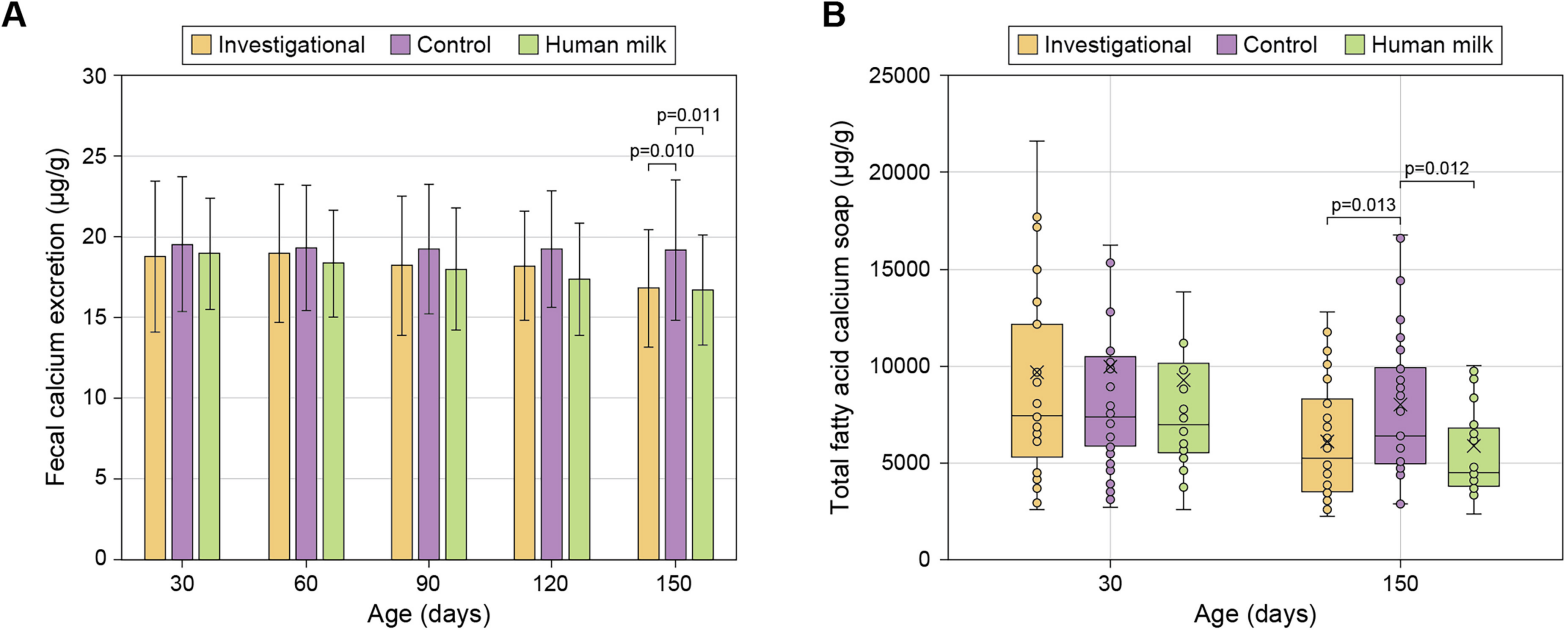
Fecal calcium excretion **(A)** and total fecal fatty acid calcium soaps **(B)**. For panel **(A)**, data are shown as arithmetic means ± standard deviations. For panel **(B)**, data are shown as medians (horizontal bar), IQRs (box), ranges (whiskers); arithmetic means are also indicated (cross). See Table 1 for sample sizes.

At 150 days, both the Investigational and HM groups showed comparable levels of calcium excretion that were significantly lower than those observed in the Control group (12% and 13% lower for Investigational and HM groups, respectively). Earlier time points (30–120 days) showed no group differences (Figure 2A and Table 1).

Similarly, at 150 days, fecal fatty acid calcium soaps were similar in the Investigational and HM groups, but significantly reduced relative to the control (21% and 31% lower for Investigational and HM groups, respectively). At 30 days, there were no significant differences in total fatty acid calcium soap levels among the groups (Figure 2B and Table 1).

### 3.4 Hair trace elements

**FIGURE 3:**
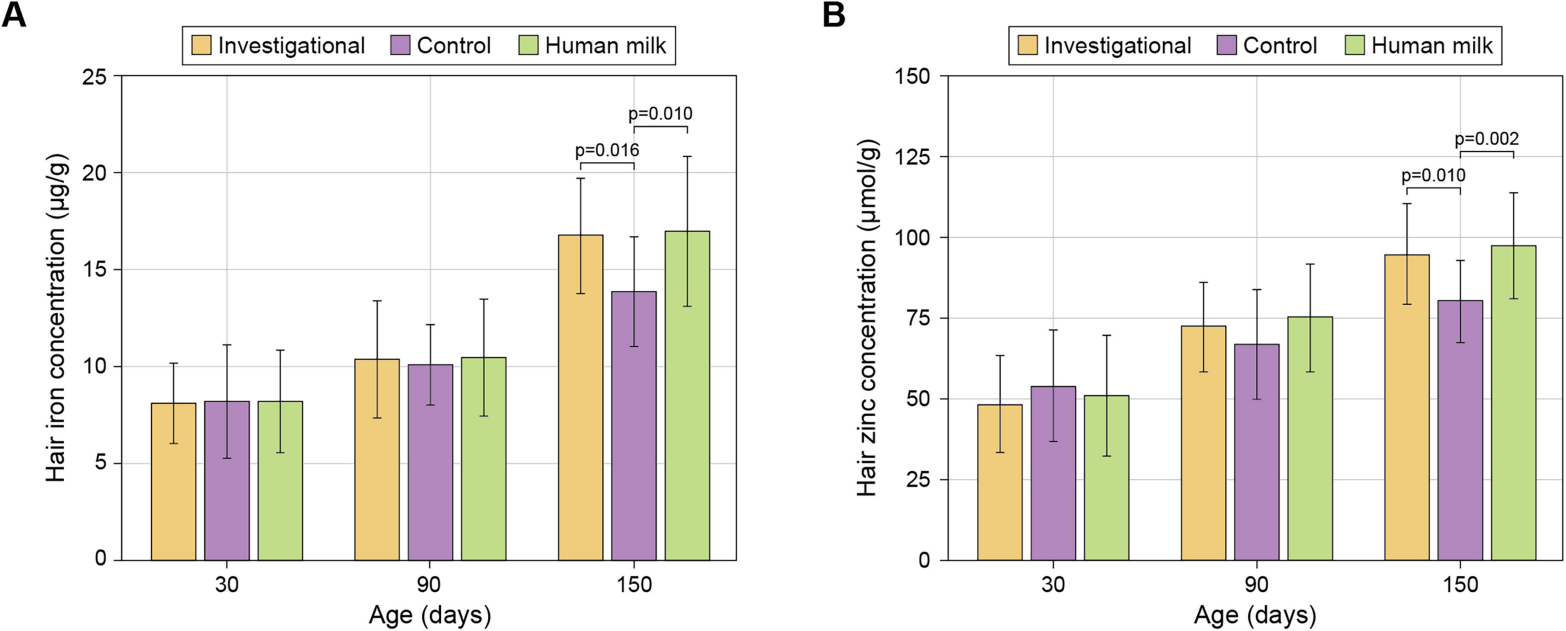
Iron **(A)** and zinc **(B)** concentrations in hair. Data are shown as arithmetic means ± standard deviations. See Table 1 for sample sizes.

Hair levels of iron and zinc increased gradually from 30 to 150 days. At 150 days, the Investigational and HM groups had similar levels of iron and zinc, that were significantly higher than the levels in the Control group (21% and 23% higher iron, and 18% and 22% higher zinc for Investigational and HM groups, respectively). Differences were not significant at earlier time points (Figure 3 and Table 1).

### 3.5 Stress and immunity markers

Cortisol and TNF-α were measured as biomarkers of stress and immune response to provide biochemical data consistent with the gut comfort effects observed with A2 milk-based IF (19). At 150 days, cortisol levels were comparable in the Investigational and HM groups, but significantly lower compared to the Control group (5% lower for both Investigational and HM groups; Table 1). There was a trend toward lower values also in TNF-α levels, but the differences were not significant.

## 4 Discussion

We have previously reported that an infant formula based on A2 milk and containing high sn-2 palmitate levels and CPP resulted in significantly greater body length, greater growth rate in head circumference, larger daily stool frequency, softer stools, higher bone quality measured by ultrasound, fewer hours of crying, less abdominal distention, burps, and flatus, and less constipation. Furthermore, those parameters were comparable to those measured in HM-fed infants (15). The present study complements those findings and provides biochemical evidence, from analyses pre-specified in the study protocol, that supports the observed clinical benefits through the analysis of biological samples collected during that trial.

A key aspect of this work is the study of an IF combining three ingredients thought to work in concert. While these types of IFs are common in the market, they are rarely studied in randomized, double-blind, controlled trials. While previous research has explored the effects of sn-2 palmitate (6, 7, 10, 11, 13, 35), CPP (29), or A2 β-casein individually, our study evaluated their combined impact to provide real-life data for physicians and healthcare professionals supporting parents in the difficult choice of an IF when breastfeeding is not an option.

### 4.1 Gut health and gastrointestinal comfort

At 150 days, infants in both the Investigational and HM groups exhibited significantly lower fecal concentrations of stearic acid, palmitic acid, and total fatty acid calcium soaps compared with the Control group fed with a standard IF. This reduction likely stems from the enhanced absorption of long-chain saturated fatty acids, leading to reduced formation of insoluble fatty acid calcium soaps in the gut, with the consequent enhanced absorption of calcium. These findings are consistent with previous studies showing higher long-chain saturated fatty acid absorption with IFs containing high sn-2 palmitate (6, 7, 10, 11, 13, 35). Palmitate at the sn-2 position of triacylglycerols remains as a 2-monoglyceride after the action of gastrointestinal lipases. The 2-monoglyceride of palmitate is absorbed more efficiently than the free palmitate resulting from lipase action at sn-1 and sn-3 positions (36). Saturated fatty acids released from sn-1 and sn-3 positions are free to form calcium soaps in the lumen of the intestine. These soaps, on top of sequestering calcium, precipitate hardening the stool. Harder stools reduce stool frequency, cause gut discomfort and constipation which, in infants, lead to more crying and interrupted sleep (10, 11, 14, 15, 37, 38).

It is worth noting that some previous studies detected the positive effects of sn-2 palmitate fortification at earlier time points (6–10). These studies are designed to remove intra-subject variability by measuring nutrients input and output (balance studies) or employing cross-over clinical study designs. Our study tries to replicate real-life feeding conditions by recruiting subjects at around 30 days of age with variable pre-study feeding patterns. In fact, for both Investigational and Control groups, around half of the infants were fed formulas supplemented with sn-2 palmitate prior to the study (15). With such a variable starting point, the differences we observe are likely underlying at earlier time points, as indicated by the medians (Table 1), but become statistically resolvable at 150 days. Indeed, comparable studies that recruited healthy infants of similar age have struggled to measure early effects consistently (39–41).

### 4.2 Infant growth and bone health

Calcium excretion was significantly lower in the Investigational and HM groups compared with the Control. This, together with the reduction in calcium soaps formation, suggests improved calcium absorption (6, 8). Previous studies indicated that improved calcium availability can enhance bone health indices associated with mass and strength in infants fed with formulas containing high levels of sn-2 palmitate (11, 42). CPP in the investigational formula may have provided additional support to the effect of high sn-2 palmitate, in line with research showing that CPP improves calcium absorption and bone mineralization (29, 30, 43–45).

Zinc and iron are essential micronutrients that support growth, immune function, and cognitive development in infants. Their addition to infant formula ensures that babies receive adequate amounts of these nutrients, particularly when breastfeeding is not an option (22, 46). Our study showed progressive increases in iron and zinc across all groups from 30 to 150 days. By the end of the study, infants in the Control group had significantly lower hair iron and zinc levels compared to those in the HM and Investigational groups. Unlike serum measurements, which reflect acute fluctuations, hair mineral content serves as a proxy for long-term mineral status. Given that only non-invasive techniques were available during our study, the hair mineral content effect should be interpreted with caution. However, higher calcium, iron, and zinc bioavailability with CPP has been previously shown *in vitro*, in animals, and human trials (29–31, 43–45, 47–49).

Together with enhanced fatty acids, i.e., energy, absorption, an increased mineral status is consistent with the positive clinical effects observed on infant growth and bone health (15).

### 4.3 Stress and immunity

Salivary cortisol has been a cornerstone in stress research for decades (50). Research shows that salivary cortisol levels in infants can reflect stress, particularly in response to acute or painful stressors (51). Infants from the Investigational and HM groups had significantly lower salivary cortisol levels than those fed with the control IF. Although TNF-α did not show significant differences, the trend went in the same direction. We interpret these results as associative and, particularly for the significant effect on cortisol, likely the reflection of the improved gastrointestinal comfort, characterized by reduced crying time, softer stools, and less abdominal distention, observed in the infants fed the investigational formula (15).

### 4.4 Limitations of the study

The principal strengths of this study lie in the use of a randomized, double-blind, controlled design with a relatively large sample size to study a real-life IF with multiple active ingredients aimed at narrowing the gap between traditional IF and HM. To our knowledge, this is the first study of this kind. Nevertheless, a key limitation should be acknowledged, i.e., the relatively short duration of the intervention and the absence of long-term follow-up limits our ability to assess sustained effects.

## 5 Conclusion

In this study, an A2 β-casein infant formula with high sn-2 palmitate and CPP showed a significant impact on reducing saturated fatty acid excretion in feces, reducing fatty acid calcium soap formation, improving calcium, iron, and zinc absorption, and regulating cortisol levels. Furthermore, these benefits were comparable in magnitude to those observed in infants fed HM. These biochemical outcomes are entirely consistent with the clinical benefits reported previously for this study (15). We propose that the clinical effects and biochemical parameters are interconnected and reflect the complementary functions of the formula’s key components. This also shows that a rational IF design that targets ingredient synergies to narrow the gap between conventional IF and HM is a valid approach to improve infant nutrition when breastfeeding is not an option.

## Data Availability

The datasets presented in this study can be found in online repositories. The names of the repository/repositories and accession number(s) can be found below: https://figshare.com/articles/dataset/Data_set_from_the_article_titled_A2_-Casein_Infant_Formula_with_High_sn-2_Palmitate_and_Casein_Phosphopeptides_CPP_Supports_Energy_and_Mineral_Absorption_in_Chinese_Infants_2025_/30865685.
